# Dodecyl-TPP Targets Mitochondria and Potently Eradicates Cancer Stem Cells (CSCs): Synergy With FDA-Approved Drugs and Natural Compounds (Vitamin C and Berberine)

**DOI:** 10.3389/fonc.2019.00615

**Published:** 2019-08-07

**Authors:** Ernestina Marianna De Francesco, Béla Ózsvári, Federica Sotgia, Michael P. Lisanti

**Affiliations:** Translational Medicine, Biomedical Research Centre, School of Environment and Life Sciences, University of Salford, Greater Manchester, United Kingdom

**Keywords:** Tri-Phenyl-Phosphonium (TPP), Cancer Stem Cells (CSCs), mitochondria, cancer therapy, vitamin C, Doxycycline, cancer metabolism, metabolic plasticity

## Abstract

Elevated mitochondrial biogenesis and/or metabolism are distinguishing features of cancer cells, as well as Cancer Stem Cells (CSCs), which are involved in tumor initiation, metastatic dissemination, and therapy resistance. In fact, mitochondria-impairing agents can be used to hamper CSCs maintenance and propagation, toward better control of neoplastic disease. Tri-Phenyl-Phosphonium (TPP)-based mitochondrially-targeted compounds are small non-toxic and biologically active molecules that are delivered to and accumulated within the mitochondria of living cells. Therefore, TPP-derivatives may represent potentially “powerful” candidates to block CSCs. Here, we evaluate the metabolic and biological effects induced by the TPP-derivative, termed Dodecyl-TPP (d-TPP) on breast cancer cells. By employing the 3D mammosphere assay in MCF-7 cells, we demonstrate that treatment with d-TPP dose-dependently inhibits the propagation of breast CSCs in suspension. Also, d-TPP targets adherent “bulk” cancer cells, by decreasing MCF-7 cell viability. The analysis of metabolic flux using Seahorse Xfe96 revealed that d-TPP potently inhibits the mitochondrial oxygen consumption rate (OCR), while simultaneously shifting cell metabolism toward glycolysis. Thereafter, we exploited this ATP depletion phenotype and strict metabolic dependency on glycolysis to eradicate the residual glycolytic CSC population, by using additional metabolic stressors. More specifically, we applied a combination strategy based on treatment with d-TPP, in the presence of a selected panel of natural and synthetic compounds, some of which are FDA-approved, that are known to behave as glycolysis (Vitamin C, 2-Deoxy-Glucose) and OXPHOS (Doxycyline, Niclosamide, Berberine) inhibitors. This two-hit scheme effectively decreased CSC propagation, at concentrations of d-TPP toxic only for cancer cells, but not for normal cells, as evidenced using normal human fibroblasts (hTERT-BJ1) as a reference point. Taken together, d-TPP halts CSCs propagation and targets “bulk” cancer cells, without eliciting the relevant undesirable off-target effects in normal cells. These observations pave the way for further exploring the potential of TPP-based derivatives in cancer therapy. Moreover, TPP-based compounds should be investigated for their potential to discriminate between “normal” and “malignant” mitochondria, suggesting that distinct biochemical, and metabolic changes in these organelles could precede specific normal or pathological phenotypes. Lastly, our data validate the manipulation of the energetic machinery as useful tool to eradicate CSCs.

## Introduction

Intact and enhanced metabolic function are necessary to support the elevated bioenergetic and biosynthetic demands of cancer cells toward tumor growth and metastatic dissemination (1, 2). Not surprisingly, mitochondrially-dependent metabolic pathways provide an essential biochemical platform for cancer cells, by extracting energy from several distinct fuels sources (3).

Recently, energetic metabolism and mitochondrial function have been linked to certain dynamics involved in the maintenance and propagation of Cancer Stem Cells (CSCs), which are a distinct cell sub-population, within the tumor mass, involved in tumor initiation, metastatic spread and resistance to anti-cancer therapies (3–9). For instance, CSCs show a peculiar and unique increase in mitochondrial mass, as well as enhanced mitochondrial biogenesis and higher activation of mitochondrial protein translation, suggestive of a strict reliance on mitochondrial function (10–13).

Consistent with these observations, elevated mitochondrial metabolic function, and OXPHOS have been detected in CSCs, across multiple tumor types (10–13). Likewise, the fluorescent mitochondrial dye, MitoTracker, has been used to enrich and purify CSCs, due to its ability to accumulate in and distinguish between different cancer cell sub-populations, characterized by a higher degree of anchorage-independent growth and higher tumor-initiating capability (10). During asymmetric cell division, “newly-synthesized” mitochondria are clustered within daughter cells, that retain stem-like phenotype. In contrast, daughter cells committed to differentiation, progressively loose stem traits because they receive older, less-efficient mitochondria. This observation further supports the idea that the most functionally viable and undamaged mitochondria, are selected for supporting stemness traits in cancer cells (14).

Based on these observations, novel pharmacological approaches aimed at targeting mitochondria in CSCs have been proposed and successfully applied in pre-clinical and clinical studies (15–23). For instance, the antibiotic Doxycycline, which decreases mitochondrial protein translation as an off-target effect, has been suggested for repurposing in the clinical management of early breast cancer patients for its ability to selectively target CSCs (21). In addition, compounds carrying a Tri-Phenyl-Phosphonium (TPP) moiety, that serves as a Mitochondria Targeting Signal (MTS), have been shown to elicit selective inhibitory actions in cancer cells, and CSCs, but not in normal cells (24).

Nevertheless, certain limitations restrain the use of solely anti-mitochondrial agents in cancer therapy, as adaptive mechanisms can be adopted in the tumor mass, to overcome the lack of mitochondrial function (3, 25). These adaptive mechanisms include the ability of CSCs to shift from oxidative metabolism to alternate energetic pathways, in a multi-directional process of metabolic plasticity driven by both intrinsic and extrinsic factors within the tumor cells, as well as in the surrounding tumor stroma or niche (7, 26, 27). Notably, in CSCs the manipulation of such metabolic flexibility can be advantageous to enhance therapeutic efficacy (23, 26). For instance, synchronizing CSCs toward certain metabolic dependencies, thus blocking their ability to shift among several energetic pathways, may represent a useful strategy toward CSC eradication (23, 26).

Herein, we provide evidence that the TPP derivative dodecyl-TPP (d-TPP), acting as a potent inhibitor of mitochondrial function, selectively and preferentially targets cancer cells and CSCs. As a proof of concept, we show that pharmacological strategies based on the combined use of mitochondrial (i.e., d-TPP) and glycolysis inhibitors (i.e., Vitamin C), efficiently hinder CSCs propagation.

## Materials and Methods

### Materials

Dodecyl-tri-phenyl-phosphonium (d-TPP) bromide, Doxycycline, Ascorbic Acid, 2-Deoxy-D-glucose (2-DG), Berberine Chloride, and Niclosamide were all purchased from Sigma Aldrich. All compounds were dissolved in DMSO (Dimethyl Sulfoxide), except Ascorbic Acid and 2-deoxy-D-glucose (2-DG), which were dissolved in cell culture medium.

### Cell Cultures

MCF7 and MDA-MB-231 breast cancer cells were obtained from the ATCC. Human immortalized fibroblasts (hTERT-BJ1) were originally purchased from Clontech, Inc. Cells were cultured in Dulbecco's modified Eagle's medium (DMEM), supplemented with 10% FBS (fetal bovine serum), 2 mM GlutaMAX, and 1% Pen-Strep in a 37°C humidified atmosphere containing 5% CO_2_.

### Mammosphere Formation

A single cell suspension of MCF-7 or MDA-MB-231 cells was prepared using enzymatic (1x Trypsin-EDTA, Sigma Aldrich), and manual disaggregation (25 gauge needle) (28). Cells were then plated at a density of 500 cells/cm^2^ in mammosphere medium (DMEM-F12/ B27/EGF(20-ng/ml)/PenStrep) under non-adherent conditions, in culture dishes coated with (2-hydroxyethylmethacrylate) (poly-HEMA, Sigma Aldrich), in the presence of treatments, as required. Cells were grown for 5 days and maintained in a humidified incubator at 37°C at an atmospheric pressure in 5% (v/v) carbon dioxide/air mixture. After 5 days for culture, spheres >50 μm were counted using an eye-piece (graticule), and the percentage of cells plated which formed spheres was calculated and is referred to as percentage mammosphere formation. Mammosphere assays were performed in triplicate and repeated three times independently.

### Seahorse XFe-96 Metabolic Flux Analysis

Extracellular acidification rates (ECAR) and real-time oxygen consumption rates (OCR) for MCF-7 cells were determined using the Seahorse Extracellular Flux (XFe-96) analyzer (Seahorse Bioscience) (11). Briefly, 15,000 MCF-7 cells per well were seeded into XFe-96 well cell culture plates, and incubated overnight to allow attachment. Cells were then treated with increasing concentrations of d-TPP (50–500 nM) for 24 h. Vehicle alone (DMSO) control cells were processed in parallel. Then, cells were washed in pre-warmed XF assay media (or for OCR measurement, XF assay media supplemented with 10 mM glucose, 1 mM Pyruvate, 2 mM L-glutamine, and adjusted at 7.4 pH). Cells were then maintained in 175 μL/well of XF assay media at 37C, in a non-CO_2_ incubator for 1 h. During the incubation time, 5 μL of 80 mM glucose, 9 μM oligomycin, and 1 M 2-deoxyglucose (for ECAR measurement) or 10 μM oligomycin, 9 μM FCCP, 10 μM Rotenone, 10 μM antimycin A (for OCR measurement), were loaded in XF assay media into the injection ports in the XFe-96 sensor cartridge. Data sets was analyzed using XFe-96 software and afterwards the measurements were normalized by protein content (via the SRB assay). All experiments were performed three times independently.

### Sulphorhodamine B Assay

Protein content in viable cells was assessed in MCF-7 and hTERT-BJ1 cells, by using the sulphorhodamine (SRB) assay, based on the measurement of cellular protein content. After treatment with d-TPP (50–1 μM) for 24, 48, or 72 h, cells were fixed with 10% trichloroacetic acid (TCA) for 1 h in the cold room, and dried overnight at room temperature. Then, cells were incubated with SRB for 15 min, washed twice with 1% acetic acid, and air dried for at least 1 h. Finally, the protein-bound dye was dissolved in a 10 mM Tris pH 8.8 solution and read using a plate reader at 540 nm.

### xCELLigence RTCA System (ACEA Biosciences Inc.)

The xCELLigence RTCA system provides a useful approach for the real-time monitoring of the biological status of adherent cells, by measuring their electrical impedance, expressed as a cell index (CI) value. Five thousand MCF-7 cells were seeded in a 16-well plate (E-plate). Twenty-four hours after seeding, cells were treated with vehicle or increasing concentrations of d-TPP (from 50 to 250 nM) for additional 72 h. Real-time cell-analysis (RTCA) was performed by measuring the cell-induced electrical impedance values, which were automatically recorded every 15 min for 96 h. This approach allows the quantification of the onset and kinetics of the cellular response. Experiments were repeated three times independently, using quadruplicate samples for each condition.

### Statistical Analysis

Data is represented as the mean ± standard error of the mean (SEM), taken over ≥3 independent experiments, with ≥3 technical replicates per experiment, unless otherwise stated. Statistical significance was measured, using the *t*-test. The *p* ≤ 0.05 was considered to be statistically significant.

## Results

We have recently established that compounds carrying a (TPP) moiety inhibit mitochondrial function and, therefore, reduce CSC propagation (24). More specifically, using an ATP (Adenosine Triphosphate) depletion assay as a surrogate marker of mitochondrial dysfunction, we identified, among 9 TPP-derivatives subjected to screening, the compound 2-butene-1,4-bis-TPP (b-TPP), as the most effective in inhibiting CSC propagation (IC-50 ~ 500 nM) (24). The TPP moiety, which acts as a mitochondrial targeting signal (MTS), is chemically attached to a cargo molecule via a covalent bond. The intrinsic nature of the cargo molecule and its binding to the TPP structure may deeply influence the accumulation of the TPP-derivative in the mitochondria of living cells, thereafter impacting its overall biological function.

Herein, we characterized the metabolic and biological properties of dodecyl-TPP (d-TPP), establishing a combination strategy that efficiently targets the energetic cell machinery of CSCs to achieve their eradication.

### d-TPP Inhibits CSC Propagation

**Figure 1:**
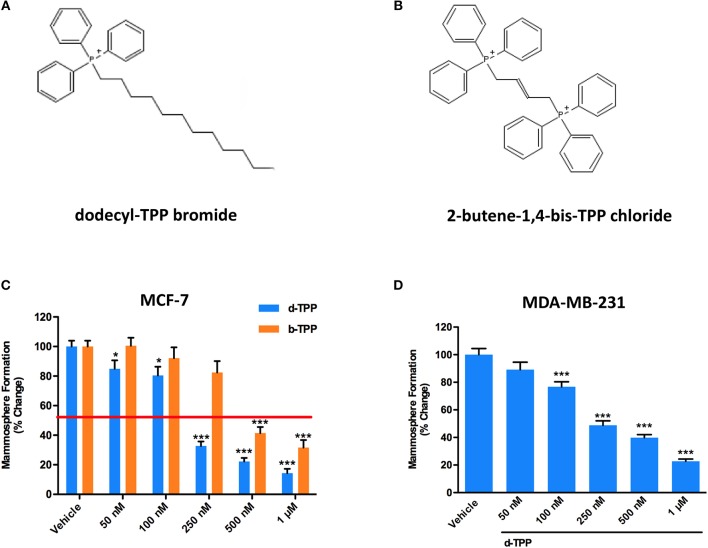
d-TPP dose-dependently inhibits mammosphere formation in both ER(+) and triple-negative cell lines. **(A,B)** Structures of the TPP derivatives d-TPP (dodecyl-TPP) and b-TPP (2-butene-1,4-bis-TPP). **(C,D)** Differential inhibition of the mammosphere-forming activity of MCF-7 and MDA-MB-231 breast CSCs, after treatment with d-TPP (blue) and b-TPP (orange). Cells were treated for 5 days in mammosphere media with vehicle alone (DMSO) or increasing concentrations of the drugs (50 nM−1 μM). Note that d-TPP is approximately twice as potent as b-TPP in inhibiting mammosphere formation, with an IC-50 of approximately 250 nM. Data shown are the mean ± SEM of at least three independent experiments performed in triplicate. ^*^p < 0.05; ^***^p < 0.001 indicates significance, all relative to the vehicle-alone treated cells.

Figure 1A shows the structure of d-TPP, which contains a long hydrophobic saturated aliphatic chain with 12 carbons, covalently-linked to a TPP moiety. Figure 1B shows the structure of b-TPP, which contains two TPP moieties. In contrast, note that d-TPP has only one TPP moiety.

Firstly, we performed the mammosphere formation assay as a read-out for CSC propagation, in the Estrogen Receptor (ER)-positive MCF-7 breast cancer cells, treated with increasing concentration of d-TPP. As shown in Figure 1C, treatment with d-TPP resulted in an 80% reduction in CSC activity at the highest concentrations tested (500 nM and 1 μM); a slight (20%), but significant reduction, in mammosphere formation was already observed at very low concentrations (50 and 100 nM). In a parallel analysis of CSC activity, d-TPP was more than twice as potent as b-TPP (Figure 1C). A similar inhibitory trend for CSC propagation was observed in the triple-negative breast cancer cells line MDA-MB-231 (Figure 1D).

**Figure 2:**
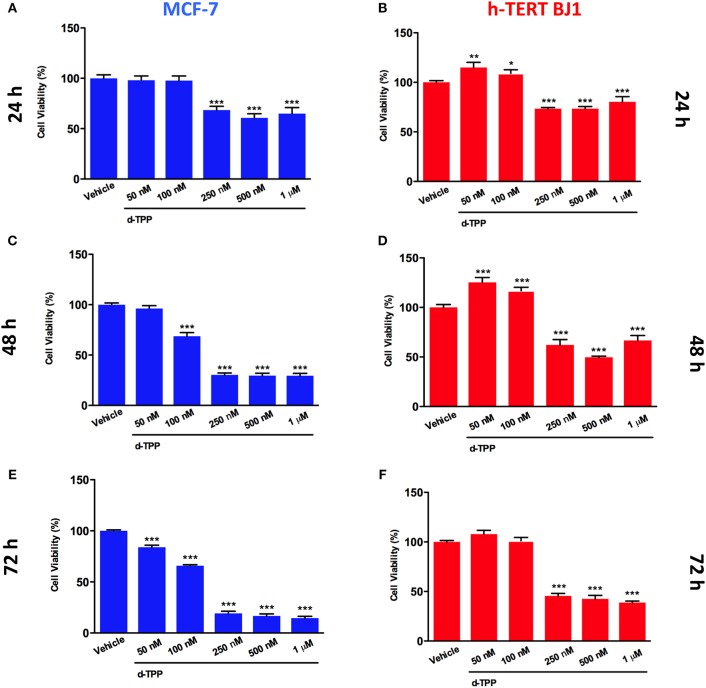
Effects of d-TPP on cell viability. Evaluation of cell viability using the SBR assay in MCF-7 **(A,C,E)** and h-TERT BJ1 **(B,D,F)** cells treated with increasing concentrations of d-TPP (50 nM−1 μM) for 24, 48, and 72 h, as indicated. Data shown are the mean ± SEM of at least three independent experiments performed in triplicate. ^*^p < 0.05; ^**^p < 0.01; ^***^p < 0.001 indicates significance, all relative to the vehicle-alone treated cells.

Our previous study has indicated that certain TPP-derivatives may interfere with mitochondrial function in cancer cells, impairing their metabolic activity and ultimately leading to a reduction in cancer cell viability. Of interest, the actions of certain TPP-compounds are selectively elicited on cancer cells, but not on normal cells (24). Thereafter, we tested the ability of d-TPP to decrease cell viability in MCF-7 breast cancer cells treated with increasing concentrations of d-TPP (from 50 nM to 1 μM), over different period of time (from 24 to 72 h). A similar experimental approach was used in normal human fibroblasts (hTERT-BJ1). As shown in Figure 2, d-TPP reduced MCF-7 cell viability in a time- and dose-dependent manner. The highest concentrations of the compound (250–500 nM and 1 μM), led to early reductions in cell viability (nearly 30%), which was evident already after 24 h of treatment. After 72 h of treatment, the reduction in cell viability was nearly 80% for the highest concentrations (250–500 nM and 1 μM) and a slight (nearly 30%) but significant inhibitory action was detected also at the lowest concentrations (50 and 100 nM).

On the other hand, in hTERT-BJ1 cells only the highest concentrations of the compound (250–500 nM and 1 μM) led to a reduction in cell viability, whereas the lowest concentrations (50 and 100 nM) showed little or no toxicity (Figures 2B–F). These results support the idea that d-TPP could be selectively used to target cancer cells, rather than normal cells, when used at low concentrations.

### d-TPP Inhibits OXPHOS and Activates Glycolysis Toward the Acquisition of Metabolic Inflexibility

In cancer cells, the intrinsic ability to flexibly shift from one fuel source to another, according to the local availability, is a pre-requisite for abnormally high proliferation, cell survival, and metastatic dissemination to distant sites (26, 27, 29). Any pharmacological or metabolic approach aimed at compromising this flexibility in the energetic cancer cell machinery will negatively impact tumor progression [reviewed in (26)].

**Figure 3:**
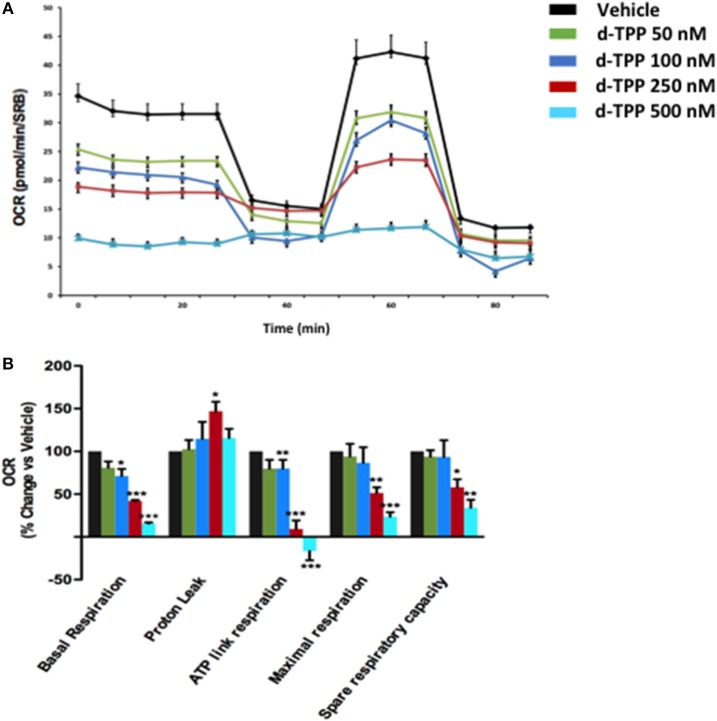
Mitochondrial respiration is inhibited in MCF-7 cells treated with d-TPP. The metabolic profile of MCF-7 cells treated with increasing concentrations of d-TPP (50–500 nM) was assessed using the Seahorse XFe96 analyzer. **(A)** Representative tracings of metabolic flux. Dose-dependent significant reductions in basal respiration, maximal respiration, ATP levels, and spare respiratory capacity were observed **(B)**. Data shown are the mean ± SEM of 3 independent experiments performed in sextuplicate. ^*^p < 0.05; ^**^p < 0.01; ^***^p < 0.001 indicates significance, all relative to the vehicle-alone treated cells.

To understand the effects of d-TPP treatment on cancer cell metabolism, we performed metabolic flux analysis using the Seahorse XFe96. In MCF-7 cells, a dramatic reduction in (OCR) was observed after 24 h of treatment, with increasing concentrations of d-TPP (Figure 3). Basal mitochondrial respiration was reduced, with an IC-50 of ~250 nM (the same concentration of the d-TPP necessary to halve CSC activity); likewise, ATP levels were also dose-dependently depleted.

**Figure 4:**
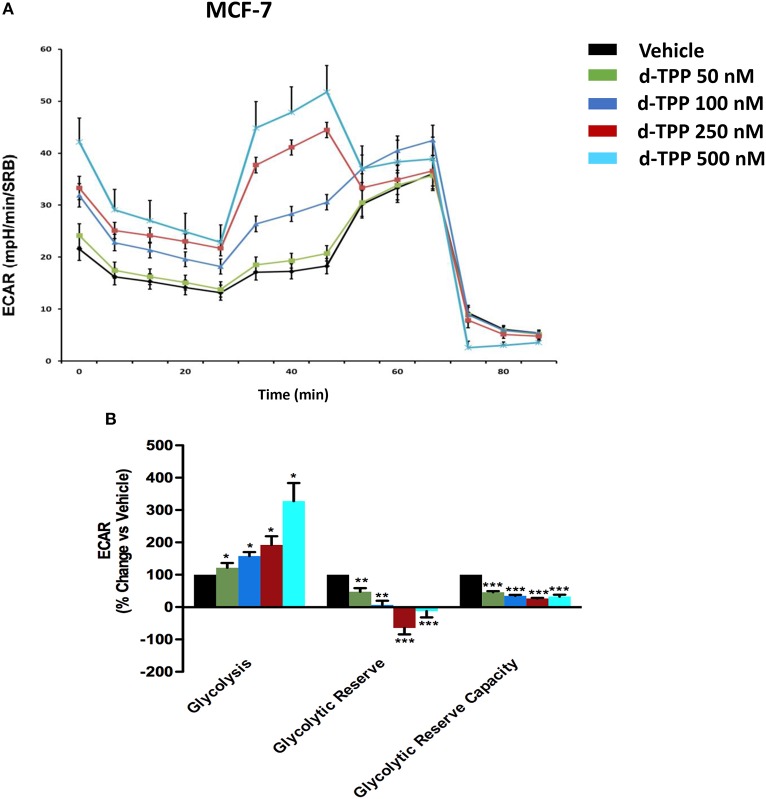
Glycolysis is increased in MCF-7 cells treated with d-TPP. The metabolic profile of MCF-7 cells treated with increasing concentrations of d-TPP (50 nM to 500 nM) was assessed using the Seahorse XFe96 analyzer. **(A)** Representative tracings of metabolic flux. **(B)** Dose-dependent significant increases in glycolysis and decreases in glycolytic reserve, as well as glycolytic reserve capacity, were observed. Data shown are the mean ± SEM of three independent experiments performed in sextuplicate. ^*^p < 0.05; ^**^p < 0.01; ^***^p < 0.001 indicates significance, all relative to the vehicle-alone treated cells.

Strikingly, the opposite trend was observed for glycolysis, which was substantially and dose-dependently increased, as indicated by the analysis of ECAR (extracellular acidification rate) in MCF-7 cells treated with d-TPP (Figure 4).

Taken together, these data indicate that d-TPP impairs mitochondrial function, thereafter compromising the ability to obtain ATP from oxidative phosphorylation. In order to cope with this stressful metabolic setting, cancer cells are forced toward a purely glycolytic phenotype, more strictly depending on glucose to fulfill their high energetic demands. In this scenario, the anti-mitochondrial effect elicited by d-TPP treatment serves as a functional metabolic synchronizer toward an obligated and inflexible glycolytic dependence.

### Using d-TPP in a “Two-Hit” Metabolic Strategy to Potently Target CSCs Vulnerabilities

As we previously observed, this scenario of energetic inflexibility can be further exploited to metabolically weaken CSCs, by the addition of other metabolic stressors (23, 26). Therefore, we performed 3D mammosphere assays using d-TPP in combination with selected glycolysis and OXPHOS inhibitors, of both synthetic and natural origin, including two FDA approved drugs, as detailed below.

This drug combination strategy has been designed as a “two-hit” scheme, where the first metabolic hit is represented by d-TPP and the second metabolic hit is represented by either Vitamin C, 2-deoxy-glucose, Doxycycline, Niclosamide, or Berberine Chloride.

For this set of experiments, we selected a concentration of d-TPP equal to 100 nM, which is the dose of the chemical selectively toxic only to cancer cells, but not normal cells (Figure 2).

**Figure 5:**
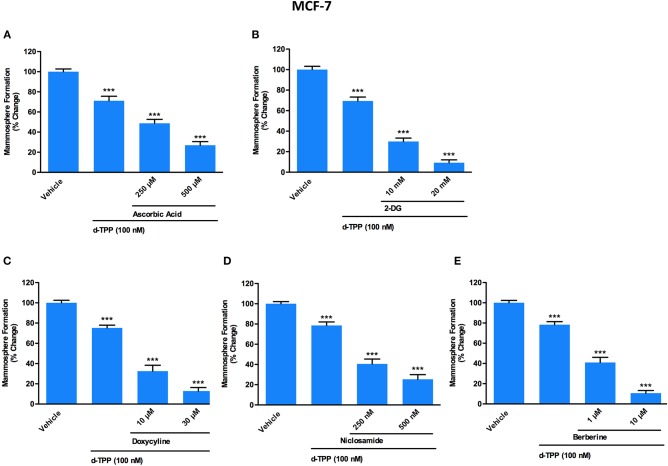
A panel of clinically-approved drugs, natural compounds and synthetic chemicals, interfering with cell metabolism, potentiates d-TPP effects on mammosphere formation. Evaluation of mammosphere formation in MCF-7 cells cultured in low attachment plates and treated with Vehicle or d-TPP (100 nM), alone or in combination with the glycolysis inhibitors Ascorbic Acid (250 and 500 μM) **(A)**, 2-deoxy-glucose (2-DG) (10 and 20 mM) **(B)**; the OXPHOS inhibitors Doxycycline (10 and 30 μM) **(C)**, Niclosamide (250 and 500 nM) **(D)**, and Berberine (1 and 10 μM) **(E)**. Data shown are the mean ± SEM of at least three independent experiments performed in triplicate ^***^p < 0.001 indicates significance, all relative to the vehicle-alone treated cells.

Of note, a natural (Vitamin C) as well as a synthetic (2-deoxy-glucose, 2-DG) glycolysis inhibitor were able to potentiate the inhibitory effect of d-TPP on CSC activity (Figures 5A,B). In particular, treatment with Vitamin C inhibited the propagation of CSCs by >50% at 250 μM and >70% at 500 μM, when used in combination with d-TPP (100 nM) (Figure 5A). Previously, we showed that the IC-50 for Vitamin C is 1 mM for MCF-7 CSC propagation (13). Therefore, treatment with d-TPP confers an approximate 4-fold increase in CSC sensitivity to Vitamin C.

The inhibitory effect of 2-DG was observed already at the concentration of 10 mM (which conferred a 2-fold increase in the inhibitory effect of d-TPP alone) and was more dramatic at 20 mM (almost completely suppressing CSC activity, with <10% residual mammosphere formation capability) (Figure 5B).

Next, we used two FDA-approved drugs, namely Doxycycline and Niclosamide, as well as the natural compound Berberine, all known to behave as OXPHOS inhibitors (11, 20, 30). This strategy is based on the assumption that in cancer cells weakened by d-TPP treatment, an additional metabolic mitochondrial stressor may help to eradicate the residual CSC population.

As shown in Figure 5C, low doses of the antibiotic Doxycycline (10 μM), which is known to impair mitochondrial biogenesis and function, were sufficient to double the efficacy of d-TPP on CSC activity; this effect was potentiated in the presence of 30 μM Doxycyline. Interestingly, the anti-tapeworm drug Niclosamide, which inhibits OXPHOS, increased the efficacy of d-TPP on CSC activity by nearly 2-fold at 250 nM and by nearly 3-fold at 500 nM (Figure 5D).

Finally, we used the natural compound and OXPHOS inhibitor Berberine, the main alkaloid extracted from Coptidis rhizoma (Coptis chinensis Franch) and Phellodendri cortex (Phellodendron amurense Ruprecht), with known anti-malarial, anti-inflammatory and antibiotic activity (31). At the highest concentration tested (10 μM), Berberine inhibited CSC formation by >80% thereafter increasing the efficacy of d-TPP by almost 5-fold, nevertheless Berberine's action was evident already at 1 μM (Figure 5E).

Taken together, these findings suggest that a mitochondrial-impairing agent like d-TPP compromises the normal functioning of the energetic cancer cell machinery, thus significantly narrowing the possibility to flexibly use alternate fuels and metabolic pathways. This has a negative impact on CSC biology and propagation, as well as cancer cell viability and proliferation. The kinetics of d-TPP action on the proliferation of adherent MCF-7 cells was independently evaluated using the xCELLigence system that allows the real-time, label-free, monitoring of cell health and behavior, by measuring electrical impedance, whose magnitude is strictly dependent on the number of cells.

**Figure 6:**
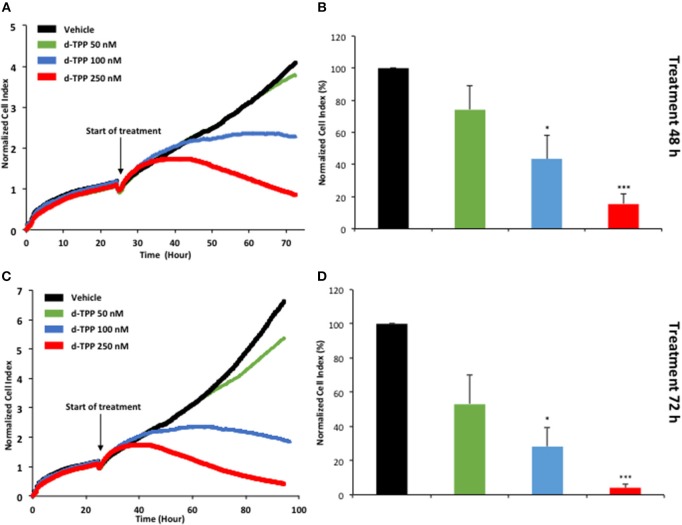
Real-time profiling of cell viability using the xCELLigence system. The xCELLigence system allows for the real-time, label-free, monitoring of cell health, and behavior, via high frequency measurement of cell-induced electrical impedance. Five-thousand MCF-7 cells were seeded in a 16-well E-plate. Twenty-four hours after seeding, cells were treated with increasing concentrations of d-TPP (50–250 nM) for further 72 h. **(A,C)** Representative cell tracings of the impedance profile indicating the normalized cell index is shown at 48 h **(A)** and 72 h **(C)**. **(B,D)** The graphs show cell index values, normalized at the time of d-TPP stimulation, reached after 48 **(B)** or 72 h **(D)** of d-TPP treatment. Data shown are the mean ± SEM of at least three independent experiments performed in triplicate. ^*^p < 0.05; ^***^p < 0.001 indicates significance, all relative to the vehicle-alone treated cells.

As shown in Figure 6, the effects of d-TPP on MCF-7 cells are dose- and time-dependent, with a trend toward reduced cell number after 72 h of treatment, even with the lowest dose tested (50 nM d-TPP). At higher doses (100 nM), a major cytostatic effect was detected, whereas clear cytotoxicity was observed at the concentration of 250 nM.

## Discussion

In this report, we provide solid evidence to validate the use of an integrated metabolic strategy to eradicate CSCs. More specifically, we demonstrate that in breast cancer cells the compound d-TPP, which carries a mitochondria-targeting (TPP) motif, inhibits mitochondrial function, conferring a dose and time-dependent reduction in cell viability, as well as inhibiting the formation of 3D mammospheres, assayed as a read-out for CSC activity. Quantitatively similar results were obtained with both ER(+) [MCF7] and Triple Negative [MDA-MB-231] cell lines.

The analysis of metabolic flux revealed that d-TPP potently inhibits mitochondrial basal respiration, as well as ATP production, thereby providing a rational for the reduced functional capability observed in response to the treatment. As a consequence, d-TPP-treated breast cancer cells preferentially exhibit a glycolytic phenotype, which allows them to cope with the anti-mitochondrial effects elicited by d-TPP. Despite the acquisition of this compensatory glycolytic phenotype, d-TPP treatment weakens CSCs, rendering them more sensitive to the action of a second metabolic inhibitor, as evidenced using the glycolysis inhibitors Vitamin C and 2-DG, as well as the OXPHOS inhibitors Doxycycline, Niclosamide, and Berberine. Thus, functional validation of this approach is evidenced by the almost complete loss of CSCs activity in MCF-7 cells, simultaneously treated with d-TPP in the presence of other energetic inhibitors.

Inter- and intra-tumor heterogeneity is one of the main factors accounting for tumor progression and therapy failure (32). CSCs originate and drive such heterogeneity, due to their ability to give rise to a hierarchically differentiated progeny, which contributes toward the generation of a full repertoire of cell types within the tumor mass (33, 34). As a consequence, pharmacological strategies aimed at identifying, and selectively targeting CSCs are among the most promising, though challenging, therapeutic approaches (8, 26, 35, 36). Diverse theories have been proposed for the explanation of the origin of CSCs. According to the so-called “metabo-stemness” model, certain metabolic phenotypes may dictate the stemness properties of tumors, suggesting that specific metabolic dynamics may drive the *de novo* acquisition of stem cell traits in non-cancer or differentiated cancer cells (37, 38). Likewise, onco-metabolism has been included among the hallmarks of cancer (39). On the basis of these observations, it's not surprising that cancer metabolism has been regarded as an opportunity to selectively target CSCs (26, 40). In this regard, our laboratory and others have demonstrated that CSCs across diverse tumor types show peculiar metabolic features, for instance an increased capacity for OXPHOS, elevated mitochondrial biogenesis, as well as higher mitochondrial mass, compared to the non-stem or “bulk” cancer cell population [reviewed in (26)]. Corroborating these findings, cancer cells with higher telomerase (hTERT^high^) activity and therefore higher immortality features, exhibit an increased mitochondrial mass, compared to their hTERT^low^ counterparts (41, 42). Furthermore, a metabolic rewiring due to mitochondrial dysfunction has been shown to inhibit cancer cell proliferation, encourage cell differentiation and halt tumor growth *in vivo* (43, 44).

Clearly these observations suggest that mitochondrial-impairing agents could be used to specifically inhibit the CSC population, paving the way for the identification of chemical strategies aimed at selectively targeting mitochondria in CSCs (4, 17, 18, 45, 46). In this regard, the covalent linkage of a compound to a (TPP) cation represents a well-established method to deliver probes and imaging agents to mitochondria (47). TPP^+^ lipophilic cations may serve as very efficient chemical “vehicles” to transport small molecules to the mitochondria. In addition, their chemical synthesis is easily achievable, and the degree of accumulation in the mitochondria is elevated, because of the chemical attraction between the positively charged TPP-cation and the negative membrane potential of the mitochondrial inner membrane (47). This peculiar chemical structure would also explain at least some of the biochemical effects induced by TPP-derivatives, like the dissipation of the mitochondrial membrane potential, together with the inhibition of the respiratory chain complexes (48). Recently, we have demonstrated that the compound 2-butene-1,4-bis-TPP impairs mitochondrial metabolic function leading to the inhibition of breast CSC activity (24). The TPP-based compound tested in the current study, namely dodecyl-TPP, is at least twice as potent as b-TPP in inhibiting CSC propagation (see Figure 1C). Also, d-TPP targets the bulk of cancer cells, resulting in a time-dependent reduction in their viability. According to our data, it is conceivable to hypothesize that after short time treatment with d-TPP (24h), cells would have enough metabolic reserves to cope with the metabolic stress induced by the treatment; whereas upon stimulation with d-TPP for longer time (48–72 h), no spare metabolic fuels would be progressively available for cancer cells to survive. On the other hand, the compound has limited toxicity effect on normal fibroblasts. One of the possible explanations of this effect is that in cancer cells and CSCs, the mitochondrial membrane potential is higher than in normal cells, and this could account for the differential effects observed.

As such, TPP-based strategies would therefore be able to distinguish between “normal” and “malignant” mitochondria, mainly on the basis of the intrinsic chemical-physical characteristics of these organelles in health and disease. Of note, d-TPP treatment caused a shift in energetic metabolism toward the activation of the glycolytic pathway, which very likely represents a compensatory response to the anti-mitochondrial effects elicited by d-TPP.

**Figure 7:**
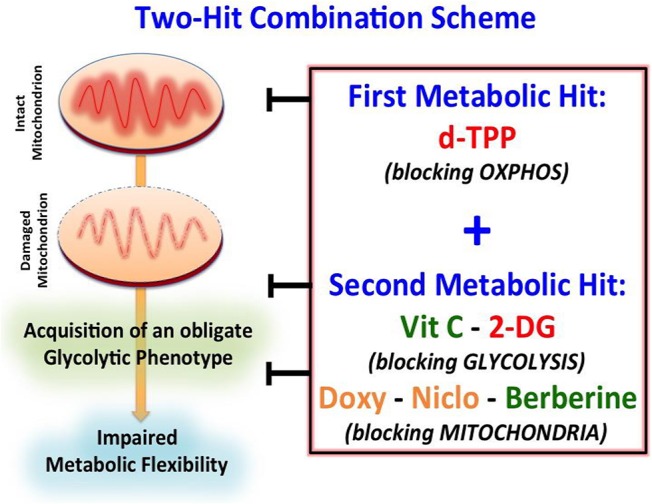
Schematic diagram showing the proposed “two-hit” metabolic strategy for eradicating CSCs. Our results suggest that CSCs may be more efficiently eradicated by using a “two-hit” metabolic strategy based on the use of a first metabolic inhibitor (i.e., d-TPP), which would weaken CSCs and their energetic machinery. The starved residual CSC population would be more sensitive to the detrimental effects of a second metabolic inhibitor (i.e., Vitamin C, Berberine, Doxycycline, Niclosamide, 2-Deoxy-Glucose). **Green**: Natural Compounds; **Orange**: FDA-approved Drugs; **Red**: Synthetic drugs.

This metabolic shift has unveiled a strict dependency of cancer cells on glycolysis after d-TPP treatment, immediately suggesting the use of a second metabolic (glycolysis or OXPHOS) inhibitor to further “starve” the residual CSCs population. This two-hit metabolic strategy had already been implemented and validated in our previous study (23). This approach is based on the use of a first metabolic inhibitor (for instance a mitochondrial-impairing agent) that serves as a first-hit, followed by the use of a second metabolic inhibitor (for instance a glycolysis or an OXPHOS inhibitor) that acts as a second-hit (Figure 7). For example, we have previously demonstrated that: (i) the antibiotic Doxycycline, acting as a mitochondrial inhibitor, impairs CSCs activity; (ii) that prolonged treatment with Doxycycline turns on the glycolytic pathway, in response to the mitochondrial dysfunction; and (iii) the use of a glycolysis inhibitor, such as Vitamin C, in combination with Doxycycline completely eradicates CSCs. These findings indicate that a combination of Doxycycline plus Vitamin C might conceivably represent a safe approach for the clinical management of cancer patients. Indeed, at biologically active concentrations Doxycycline has a very manageable spectrum of side effects, whereas Vitamin C has potentially no side effects, being used as additive to a number of preparations. Adding to this, data coming from meta-analysis of 21 published studies, as well as pre-clinical investigations have demonstrated that oral doses of vitamin C reduce cancer risk, overall mortality and disease-specific mortality in lung and breast cancer (49–52); furthermore, a 1-week oral administration of ascorbate, performed before cell inoculation, significantly decreased tumor development in a lymphoma xenograft model (53).

Clinical validation of these findings, at least for Doxycycline, has been provided in our recent pilot study, where we enrolled early breast cancer patients for a short-term (14-day) pre-operative Doxycycline administration. Notably, the analysis of post-operative vs. pre-operative breast tumor specimens showed a selective reduction in the stem markers, namely CD44 and ALDH1, in the patients receiving Doxycycline compared with controls. This provides exciting clinical evidence that mitochondrial-targeting strategies may be used to efficiently halt CSC propagation in cancer patients; nevertheless, additional studies are still necessary to investigate the action of Doxycycline together with Vitamin C, in breast cancer patients.

The data herein presented further validate the use of a metabolic “Two-Hit” approach to manipulate cancer cell metabolism, turning the metabolic plasticity of CSCs into an obligate metabolic inflexibility, which would be used to more efficiently eradicate CSCs. The potential of d-TPP to act as a safe driver of such a metabolic shift paves the way for further investigating the role of this compound in cancer biology and bioenergetics. However, further studies are still warranted to support the use of TPP-based compounds in combination with other metabolic inhibitors, as an add-on to conventional chemotherapy.

## Data Availability

All datasets generated for this study are included in the manuscript/supplementary files.

## Author Contributions

ML and FS conceived and initiated this project. All experiments described in this paper were performed by ED and BÓ. More specifically, BÓ performed experiments of cell kinetics evaluation using the xCELLigence system. ED analyzed the data, generated the final figures, and wrote the first draft of the paper, which was then further edited by ML, FS, and BÓ.

### Conflict of Interest Statement

ML and FS hold a minority interest in Lunella Biotech, Inc. The remaining authors declare that the research was conducted in the absence of any commercial or financial relationships that could be construed as a potential conflict of interest.

## References

[1] Zhu J Thompson CB. Metabolic regulation of cell growth and proliferation. Nat Rev Mol Cell Biol. (2019) 20:436–50. 10.1038/s41580-019-0123-5 30976106 PMC6592760

[2] Pavlova NN Thompson CB. The emerging hallmarks of cancer metabolism. Cell Metabol. (2016) 23:27–47. 10.1016/j.cmet.2015.12.006 26771115 PMC4715268

[3] Vyas S Zaganjor E Haigis MC. Mitochondria and cancer. Cell. (2016) 166:555–66. 10.1016/j.cell.2016.07.002 27471965 PMC5036969

[4] Shin M-K Cheong J-H. Mitochondria-centric bioenergetic characteristics in cancer stem-like cells. Arch Pharm Res. (2019) 42:113–27. 10.1007/s12272-019-01127-y 30771209 PMC6399179

[5] Altieri DC. Mitochondrial dynamics and metastasis. Cell Mol Life Sci. (2019) 76:827–35. 10.1007/s00018-018-2961-2 30415375 PMC6559795

[6] Peiris-Pagès M Bonuccelli G Sotgia F Lisanti MP. Mitochondrial fission as a driver of stemness in tumor cells: mDIVI1 inhibits mitochondrial function, cell migration and cancer stem cell (CSC) signalling. Oncotarget. (2018) 9:13254–75. 10.18632/oncotarget.24285 29568355 PMC5862576

[7] Peiris-Pagès M Martinez-Outschoorn UE Pestell RG Sotgia F Lisanti MP. Cancer stem cell metabolism. Breast Cancer Res. (2016) 18:55. 10.1186/s13058-016-0712-6 27220421 PMC4879746

[8] Clevers H. The cancer stem cell: premises, promises and challenges. Nat Med. (2011) 17:313–9. 10.1038/nm.2304 21386835

[9] Batlle E Clevers H. Cancer stem cells revisited. Nat Med. (2017) 23:1124–34. 10.1038/nm.4409 28985214

[10] Farnie G Sotgia F Lisanti MP. High mitochondrial mass identifies a sub-population of stem-like cancer cells that are chemo-resistant. Oncotarget. (2015) 6:30472–86. 10.18632/oncotarget.5401 26421710 PMC4741545

[11] De Luca A Fiorillo M Peiris-Pagès M Ozsvari B Smith DL Sanchez-Alvarez R. Mitochondrial biogenesis is required for the anchorage-independent survival and propagation of stem-like cancer cells. Oncotarget. (2015) 6:14777–95. 10.18632/oncotarget.4401 26087310 PMC4558115

[12] Lamb R Bonuccelli G Ozsvári B Peiris-Pagès M Fiorillo M Smith DL. Mitochondrial mass, a new metabolic biomarker for stem-like cancer cells: understanding WNT/FGF-driven anabolic signaling. Oncotarget. (2015) 6:30453–71. 10.18632/oncotarget.5852 26421711 PMC4741544

[13] Bonuccelli G De Francesco EM de Boer R Tanowitz HB Lisanti MP. NADH autofluorescence, a new metabolic biomarker for cancer stem cells: identification of Vitamin C and CAPE as natural products targeting “stemness.” Oncotarget. (2017) 8:20667–78. 10.18632/oncotarget.15400 28223550 PMC5400535

[14] Katajisto P Dohla J Chaffer CL Pentinmikko N Marjanovic N Iqbal S. Asymmetric apportioning of aged mitochondria between daughter cells is required for stemness. Science. (2015) 348:340–3. 10.1126/science.1260384 25837514 PMC4405120

[15] Sotgia F Ozsvari B Fiorillo M De Francesco EM Bonuccelli G Lisanti MP. A mitochondrial based oncology platform for targeting cancer stem cells (CSCs): MITO-ONC-RX. Cell Cycle. (2018) 17:2091–100. 10.1080/15384101.2018.1515551 30257595 PMC6226227

[16] Skoda J Borankova K Jansson PJ Huang ML-H Veselska R Richardson DR. Pharmacological targeting of mitochondria in cancer stem cells: an ancient organelle at the crossroad of novel anti-cancer therapies. Pharmacol Res. (2019) 139:298–313. 10.1016/j.phrs.2018.11.020 30453033

[17] Ozsvari B Sotgia F Simmons K Trowbridge R Foster R Lisanti MP. Mitoketoscins: novel mitochondrial inhibitors for targeting ketone metabolism in cancer stem cells (CSCs). Oncotarget. (2017) 8:78340–50. 10.18632/oncotarget.21259 29108233 PMC5667966

[18] Ozsvari B Fiorillo M Bonuccelli G Cappello AR Frattaruolo L Sotgia F. Mitoriboscins: mitochondrial-based therapeutics targeting cancer stem cells (CSCs), bacteria and pathogenic yeast. Oncotarget. (2017) 8:67457–72. 10.18632/oncotarget.19084 28978045 PMC5620185

[19] Peiris-Pagès M Sotgia F Lisanti MP. Doxycycline and therapeutic targeting of the DNA damage response in cancer cells: old drug, new purpose. Oncoscience. (2015) 2:696–9. 10.18632/oncoscience.215 26425660 PMC4580062

[20] Lamb R Fiorillo M Chadwick A Ozsvari B Reeves KJ Smith DL. Doxycycline down-regulates DNA-PK and radiosensitizes tumor initiating cells: implications for more effective radiation therapy. Oncotarget. (2015) 6:14005–25. 10.18632/oncotarget.4159 26087309 PMC4546447

[21] Scatena C Roncella M Di Paolo A Aretini P Menicagli M Fanelli G. Doxycycline, an inhibitor of mitochondrial biogenesis, effectively reduces Cancer Stem Cells (CSCs) in early breast cancer patients: a clinical pilot study. Front Oncol. (2018) 8:452. 10.3389/fonc.2018.00452 30364293 PMC6194352

[22] De Francesco EM Maggiolini M Tanowitz HB Sotgia F Lisanti MP. Targeting hypoxic cancer stem cells (CSCs) with doxycycline: implications for optimizing anti-angiogenic therapy. Oncotarget. (2017) 8:56126–42. 10.18632/oncotarget.18445 28915578 PMC5593549

[23] De Francesco EM Bonuccelli G Maggiolini M Sotgia F Lisanti MP. Vitamin C and Doxycycline: a synthetic lethal combination therapy targeting metabolic flexibility in cancer stem cells (CSCs). Oncotarget. (2017) 8:67269–86. 10.18632/oncotarget.18428 28978032 PMC5620172

[24] Ozsvari B Sotgia F Lisanti MP. Exploiting mitochondrial targeting signal(s), TPP and bis-TPP, for eradicating cancer stem cells (CSCs). Aging. (2018) 10:229–40. 10.18632/aging.101384 29466249 PMC5842849

[25] Dickerson T Jauregui CE Teng Y. Friend or foe? Mitochondria as a pharmacological target in cancer treatment. Future Med Chem. (2017) 9:2197–210. 10.4155/fmc-2017-0110 29182013

[26] De Francesco EM Sotgia F Lisanti MP. Cancer stem cells (CSCs): metabolic strategies for their identification and eradication. Biochem J. (2018) 475:1611–34. 10.1042/BCJ20170164 29743249 PMC5941316

[27] Krstic J Trivanovic D Jaukovic A Santibanez JF Bugarski D. Metabolic plasticity of stem cells and macrophages in cancer. Front Immunol. (2017) 8:939. 10.3389/fimmu.2017.00939 28848547 PMC5552673

[28] Shaw FL Harrison H Spence K Ablett MP Simões BM Farnie G. A detailed mammosphere assay protocol for the quantification of breast stem cell activity. J Mammary Gland Biol Neoplasia. (2012) 17:111–7. 10.1007/s10911-012-9255-3 22665270

[29] Nakajima EC Van Houten B. Metabolic symbiosis in cancer: refocusing the Warburg lens. Mol Carcinog. (2013) 52:329–37. 10.1002/mc.21863 22228080 PMC9972501

[30] Turner N Li J-Y Gosby A To SWC Cheng Z Miyoshi H. Berberine and its more biologically available derivative, dihydroberberine, inhibit mitochondrial respiratory complex I: a mechanism for the action of berberine to activate AMP-activated protein kinase and improve insulin action. Diabetes. (2008) 57:1414–8. 10.2337/db07-1552 18285556

[31] Liu D Meng X Wu D Qiu Z Luo H. A natural isoquinoline alkaloid with antitumor activity: studies of the biological activities of berberine. Front Pharmacol. (2019) 10:9. 10.3389/fphar.2019.00009 30837865 PMC6382680

[32] Meacham CE Morrison SJ. Tumour heterogeneity and cancer cell plasticity. Nature. (2013) 501:328–37. 10.1038/nature12624 24048065 PMC4521623

[33] Prasetyanti PR Medema JP. Intra-tumor heterogeneity from a cancer stem cell perspective. Mol Cancer. (2017) 16:41. 10.1186/s12943-017-0600-4 28209166 PMC5314464

[34] Visvader JE. Cells of origin in cancer. Nature. (2011) 469:314–22. 10.1038/nature09781 21248838

[35] Apostoli AJ Ailles L. Clonal evolution and tumor-initiating cells: new dimensions in cancer patient treatment. Crit Rev Clin Lab Sci. (2016) 53:40–51. 10.3109/10408363.2015.1083944 26397062

[36] Dawood S Austin L Cristofanilli M. Cancer stem cells: implications for cancer therapy. Oncology. (2014) 28:1101–7, 1110. 25510809

[37] Menendez JA Alarcón T. Metabostemness: a new cancer hallmark. Front Oncol. (2014) 4:262. 10.3389/fonc.2014.00262 25325014 PMC4179679

[38] Menendez JA. The metaboloepigenetic dimension of cancer stem cells: evaluating the market potential for new metabostemness-targeting oncology drugs. Curr Pharm Des. (2015) 21:3644–53. 10.2174/1381612821666150710150327 26166605

[39] Hanahan D Weinberg RA. Hallmarks of cancer: the next generation. Cell. (2011) 144:646–74. 10.1016/j.cell.2011.02.013 21376230

[40] Jagust P de Luxán-Delgado B Parejo-Alonso B Sancho P. Metabolism-based therapeutic strategies targeting cancer stem cells. Front Pharmacol. (2019) 10:203. 10.3389/fphar.2019.00203 30967773 PMC6438930

[41] Lamb R Ozsvari B Bonuccelli G Smith DL Pestell RG Martinez-Outschoorn UE. Dissecting tumor metabolic heterogeneity: telomerase and large cell size metabolically define a sub-population of stem-like, mitochondrial-rich, cancer cells. Oncotarget. (2015) 6:21892–905. 10.18632/oncotarget.5260 26323205 PMC4673134

[42] Bonuccelli G Peiris-Pages M Ozsvari B Martinez-Outschoorn UE Sotgia F Lisanti MP. Targeting cancer stem cell propagation with palbociclib, a CDK4/6 inhibitor: telomerase drives tumor cell heterogeneity. Oncotarget. (2017) 8:9868–84. 10.18632/oncotarget.14196 28039467 PMC5354777

[43] Arif T Amsalem Z Shoshan-Barmatz V. Metabolic reprograming via silencing of mitochondrial VDAC1 expression encourages differentiation of cancer cells. Mol Ther Nucleic Acids. (2019) 17:24–37. 10.1016/j.omtn.2019.05.003 31195298 PMC6562189

[44] Arif T Paul A Krelin Y Shteinfer-Kuzmine A Shoshan-Barmatz V. Mitochondrial VDAC1 silencing leads to metabolic rewiring and the reprogramming of tumour cells into advanced differentiated states. Cancers (Basel). (2018) 10. 10.3390/cancers10120499 30544833 PMC6316808

[45] Zhu Y Dean AE Horikoshi N Heer C Spitz DR Gius D. Emerging evidence for targeting mitochondrial metabolic dysfunction in cancer therapy. J Clin Invest. (2018) 128:3682–91. 10.1172/JCI120844 30168803 PMC6118595

[46] Lamb R Harrison H Hulit J Smith DL Lisanti MP Sotgia F. Mitochondria as new therapeutic targets for eradicating cancer stem cells: quantitative proteomics and functional validation via MCT1/2 inhibition. Oncotarget. (2014) 5:11029–37. 10.18632/oncotarget.2789 25415228 PMC4294326

[47] Zielonka J Joseph J Sikora A Hardy M Ouari O Vasquez-Vivar J. Mitochondria-targeted triphenylphosphonium-basedcompounds: syntheses, mechanisms of action, and therapeutic and diagnostic applications. Chem Rev. (2017) 117:10043–120. 10.1021/acs.chemrev.7b00042 28654243 PMC5611849

[48] Trnka J Elkalaf M Anděl M. Lipophilic triphenylphosphonium cations inhibit mitochondrial electron transport chain and induce mitochondrial proton leak. PLoS One. (2015) 10:e0121837. 10.1371/journal.pone.0121837 25927600 PMC4415762

[49] Luo J Shen L Zheng D. Association between vitamin C intake and lung cancer: a dose-response meta-analysis. Sci Rep. (2014) 4:6161. 10.1038/srep06161 25145261 PMC5381428

[50] Harris HR Orsini N Wolk A. Vitamin C and survival among women with breast cancer: a meta-analysis. Eur J Cancer. (2014) 50:1223–31. 10.1016/j.ejca.2014.02.013 24613622

[51] Harris HR Bergkvist L Wolk A. Vitamin C intake and breast cancer mortality in a cohort of Swedish women. Br J Cancer. (2013) 109:257–64. 10.1038/bjc.2013.269 23736027 PMC3708583

[52] Ngo B Van Riper JM Cantley LC Yun J Targeting cancer vulnerabilities with high-dose vitamin C. Nat Rev Cancer. (2019) 9:271–82. 10.1038/s41568-019-0135-7 PMC6526932 30967651

[53] Gao P Zhang H Dinavahi R Li F Xiang Y Raman V. HIF-dependent antitumorigenic effect of antioxidants *in vivo*. Cancer Cell. (2007) 12:230–8. 10.1016/j.ccr.2007.08.004 17785204 PMC2084208

